# Monitoring the Systemic Human Memory B Cell Compartment of Melanoma Patients for Anti-Tumor IgG Antibodies

**DOI:** 10.1371/journal.pone.0019330

**Published:** 2011-04-29

**Authors:** Amy E. Gilbert, Panagiotis Karagiannis, Tihomir Dodev, Alexander Koers, Katie Lacy, Debra H. Josephs, Pooja Takhar, Jenny L. C. Geh, Ciaran Healy, Mark Harries, Katharine M. Acland, Sarah M. Rudman, Rebecca L. Beavil, Philip J. Blower, Andrew J. Beavil, Hannah J. Gould, James Spicer, Frank O. Nestle, Sophia N. Karagiannis

**Affiliations:** 1 Cutaneous Medicine and Immunotherapy Unit, Division of Genetics and Molecular Medicine, NIHR Biomedical Research Centre at Guy’s and St. Thomas’s Hospitals and King’s College London, King’s College London School of Medicine, St. John’s Institute of Dermatology, Guy’s Hospital, King’s College London, London, United Kingdom; 2 Randall Division of Cell and Molecular Biophysics and Division of Asthma, Allergy and Lung Biology, MRC and Asthma UK Centre for Allergic Mechanisms of Asthma, King's College London, London, United Kingdom; 3 Division of Imaging Sciences, King’s College London School of Medicine, Rayne Institute, St. Thomas's Hospital, King’s College London, London, United Kingdom; 4 Skin Tumour Unit, Guy's and St. Thomas's NHS Trust, St. John’s Institute of Dermatology, Guy’s Hospital, London, United Kingdom; 5 Clinical Oncology, Guy’s and St. Thomas’s NHS Foundation Trust, London, United Kingdom; 6 Division of Cancer Studies, Department of Academic Oncology, King’s College London, Guy's Hospital, London, United Kingdom; University of Massachusetts Medical Center, United States of America

## Abstract

Melanoma, a potentially lethal skin cancer, is widely thought to be immunogenic in nature. While there has been much focus on T cell-mediated immune responses, limited knowledge exists on the role of mature B cells. We describe an approach, including a cell-based ELISA, to evaluate mature IgG antibody responses to melanoma from human peripheral blood B cells. We observed a significant increase in antibody responses from melanoma patients (n = 10) to primary and metastatic melanoma cells compared to healthy volunteers (n = 10) (*P*<0.0001). Interestingly, we detected a significant reduction in antibody responses to melanoma with advancing disease stage in our patient cohort (n = 21) (*P*<0.0001*)*. Overall, 28% of melanoma patient-derived B cell cultures (n = 1,800) compared to 2% of cultures from healthy controls (n = 600) produced antibodies that recognized melanoma cells. Lastly, a patient-derived melanoma-specific monoclonal antibody was selected for further study. This antibody effectively killed melanoma cells *in vitro* via antibody-mediated cellular cytotoxicity. These data demonstrate the presence of a mature systemic B cell response in melanoma patients, which is reduced with disease progression, adding to previous reports of tumor-reactive antibodies in patient sera, and suggesting the merit of future work to elucidate the clinical relevance of activating humoral immune responses to cancer.

## Introduction

Malignant melanoma, the most fatal form of skin cancer, arises from malignantly-transformed melanocytes in the basal layer of the epidermis. The incidence of melanoma has been increasing at an accelerated rate in the past few decades amongst fair skinned populations [1] and advanced forms of the disease are highly resistant to treatment [2], [3]. Thus, an urgent need exists for novel therapies and earlier diagnosis.

Melanoma is widely thought to be immunogenic, supported by clinical observations such as the frequency of spontaneous tumor regressions, the prevalence of melanoma in immunosuppressed patients, and the partial success of clinically-available immune modulatory therapies such as the polyclonal immune activating cytokines IFNα-2b and IL- 2 [4], [5], [6], [7]. Host adaptive immune responses have been described in melanoma with a main focus on melanoma specific T cell responses [8], [9], and supported by successful case scenarios using immunotherapeutic strategies such as dendritic cell vaccines, adoptive T cell therapies, and CTLA4 monoclonal antibodies [7], [10], [11], [12], [13].

Limited research has focused on B cells and the specificity of antibodies they produce in cancer. Promotion of cancer development by the creation of a pro-inflammatory environment [14], [15] and anti-tumor functions by activating mature T cell responses [16] have been proposed as potential roles for B cells in animal models of cancer. While there may be host immune responses to malignancy following immunization [17], a variety of mechanisms involved in tumor escape have been described and understanding this complex relationship between immunosurveillance and tumor escape in patients is key to the design of effective immunotherapies [18], [19], [20], [21].

Despite well-characterized tumor-induced immunomodulation, immunotherapies such as monoclonal antibodies are emerging as key diagnostic and therapeutic modalities and are now standard of care for the treatment of various cancers. Antibodies for the treatment of melanoma aimed at enhancing key pathways of T cell activation (Cytotoxic T Lymphocyte-Associated Antigen 4, e.g. Ipilimumab), targeting tumor vasculature (e.g. Bevacizumab), or tumor-associated antigens (e.g. High Molecular Weight-Melanoma Associated Antigen, HMW-MAA) have demonstrated promise in clinical studies [13], [22], [23], [24]. Antibodies therefore represent an attractive approach for the treatment of melanoma.

Reports of tumor-specific antibodies in the sera of melanoma patients date back over forty years [25] and have so far provided valuable insight into immune responses to cancer. Serological studies of individuals with melanoma have shown that patients expressing certain tumor-associated antigens have antibodies against these antigens, conversely, patients without the antibodies also lack the corresponding tumor antigens [26]. These studies have been restricted to few antibodies in sera against known tumor-associated antigens. Serological studies reported IgG antibodies recognizing intracellular melanocyte and melanoma-associated antigens such as tyrosinase, tyrosinase-related protein (TRP)-1, TRP-2, and melanoma-associated glycoprotein antigen family (gp100/pmel17) in patients with melanoma. Serum-resident antibodies to some of these antigens were enhanced following polyvalent melanoma cell vaccine immunization in patients with melanoma, suggesting that melanoma-associated antigens may be immunogenic and that humoral responses to melanocyte and melanoma antigens may constitute potential targets for immunotherapy [27]. New antigens, such as the NY-ESO-1, with restricted expression in normal tissues and wide distribution in various cancers including melanoma have been discovered using serological analysis of recombinant cDNA expression libraries (SEREX) techniques tested against tumor mRNA and autologous patient sera [28]. SEREX studies from human melanomas [29] and from one cell line [30] have led to the discovery of the human testis antigen HOM-MEL-40. Many of these antigens are primarily intracellular, making them less attractive targets as monoclonal antibodies. Furthermore, serological screens may also be limited by the temporal dynamics of sera antibodies. Evaluating the reactivity of antibodies secreted by circulating B cells may therefore provide additional insight to serological evaluations by interrogating the long-term memory anti-tumor systemic mature humoral response to cancer.

The production of tumor-specific antibodies in melanoma from patient-derived B cells in the peripheral blood and tumors has been reported and has yielded a few antibodies of the IgM and IgG class [31], [32], [33], [34]. In the past, such studies have been limited by poor EBV transformation efficiency of human B cells, low production of immunoglobulin, evaluation of few patients, and lack of effective, reproducible methods to rapidly screen for tumor-specific antibodies. To address some of these limitations, we took advantage of recent advances in growing and immortalizing memory B cells in culture [35], [36], increased the number of patients evaluated, and developed a novel screening tool to specifically detect tumor-reactive antibodies against cell surface antigens on melanoma cells. Our approach entails culture of patient-derived circulating B cells and screening of the antibodies they secrete for their reactivity and specificity to melanoma cells versus melanocytes. Our strategy does not screen for antibodies against known antigens or evaluate antibodies secreted or sequestered in the serum at discrete times, but rather uniquely, the aim here is to monitor tumor cell-reactive IgG antibodies produced by B cell cultures, elucidating the breadth of the long-term mature B cell repertoire recognizing melanoma antigens expressed on the surface of cancer cells.

In this study, we screen for tumor-reactive and tumor-specific IgG antibodies produced by patient and healthy individual B cell cultures. This allowed characterization, beyond phenotype, of the circulating B cell repertoire of individuals with melanoma and clinical correlations of mature humoral responses and disease progression. We also provide an example demonstrating that this screen may facilitate the identification of antibodies able to target cancer cells.

## Results

### Detection of Tumor-specific IgG Antibodies Using a Novel Cell-based ELISA

**Figure 1:**
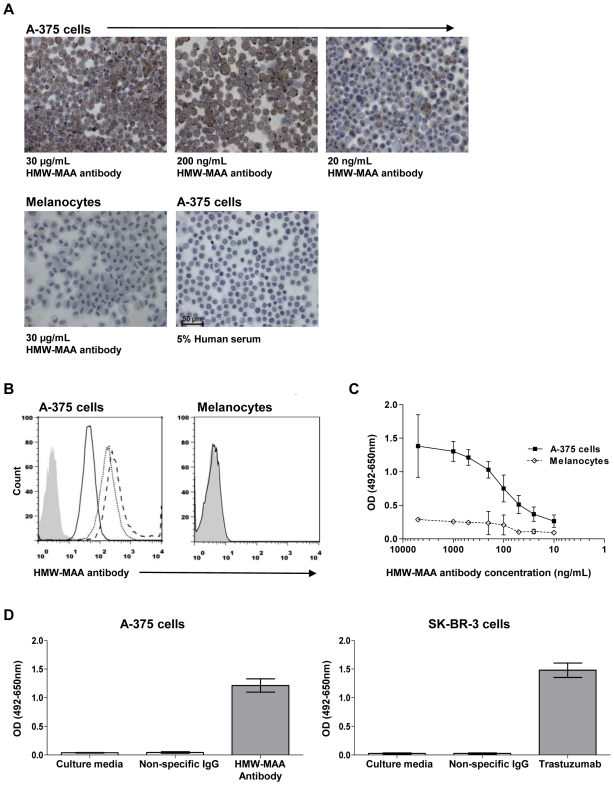
Development of a cell-based ELISA to detect tumor-specific antibodies. (**A**) Immunocytochemistry of cytospin preparations demonstrates that an antibody against the melanoma-associated antigen HMW-MAA, expressed on the surface of A-375 metastatic melanoma cells, can detect the antigen at 30 µg/mL (top left), 200 ng/mL (top middle) and 20 ng/mL (top right), while no binding to melanocytes was seen (bottom left). IgG from 5% human serum (bottom right) did not bind to A-375 cells. (**B**) Flow cytometry analysis demonstrates specific binding of the HMW-MAA antibody to A-375 cells (left) at 30 µg/mL (dashed line), 200 ng/mL (dotted line) and 20 ng/mL (solid line), but not to melanocytes (30 µg/mL HMW-MAA antibody). Isotype control is shown in shaded grey histogram. (**C**) Detection of anti-HMW-MAA antibody binding to melanoma cells over a range of antibody concentrations compared to melanocytes by cell-based ELISA. (**D**) Melanoma-specific antibody HMW-MAA binding to A-375 cells compared to an IgG isotype control or to culture media (left) utilizing the cell-based ELISA. Breast cancer-specific antibody Trastuzumab binding to SK_BR-3 cells compared to an IgG isotype control or to culture media (right). Error bars in figures represent 95% confidence intervals.

We developed and optimized a cell-based ELISA for specific detection of tumor-reactive antibodies in order to obtain a robust and optimized system for the detection of anti-tumor antibodies from patients (Figure S1). We first evaluated the sensitivity and specificity of an IgG antibody against a melanoma cell surface antigen (HMW-MAA), expressed on A-375 melanoma cells using immunocytochemistry (cytospins) and live cell flow cytometry. An anti-HMW-MAA antibody was observed to bind to A-375 cells, but not melanocytes over a range of concentrations as low as 20 ng/mL using both immunocytochemistry (Figure 1, A) and flow cytometry (Figure 1, B). Next, we compared the detection of this antibody bound to melanoma cells in our cell-based ELISA to the above methods.

Utilizing our novel ELISA, we detected tumor-specific antibodies at concentrations as low 10 ng/mL (Figure 1, C), demonstrating comparable sensitivity to flow cytometric or immunocytochemical methods. Additionally, we validated our ability to identify tumor-reactive antibodies from our patient cultures, compared to equal amounts of non-specific IgG and culture media (Figure 1, D). We also examined the potential applicability of this method to identify tumor-specific antibodies in other cancers using the mammary carcinoma cell line SK-BR-3, which highly expresses the cell surface tumor-associated antigen HER2/*neu* [37]. Trastuzumab (Herceptin™), a humanized antibody specific for HER2/*neu*, was specifically detected compared to an equal amount of a control IgG employing our method (Figure 1, D). Thus, we demonstrate that we can detect antibodies against tumor cell antigens in a sensitive, specific and reproducible manner.

### Melanoma-reactive Antibodies are More Prevalent in Melanoma Patients than Healthy Volunteers

We first established B cell cultures from the peripheral blood of melanoma patients to study antibody responses to cancer (Figure S1). In agreement with a previously published report, we detected a reduced memory B cell subset in melanoma patients. Melanoma patient and healthy volunteer B cells were cultured with B cell purity greater than 90%. Following EBV transformation and activation with a TLR9 agonist, patient B cells were observed to proliferate in culture for over eight weeks and 80% of the cells in these cultures were IgG positive. B cell cultures derived from healthy volunteers (n = 5) and melanoma patients (n = 5) had comparable mean antibody titers after 18 days, ranging from 1 to 7 µg from each individual, with an overall mean of 2.5 µg (95% CI  =  2.3 to 2.7) per culture arising from 500 B cells per well. We therefore established antibody-secreting cultures from melanoma patients with comparable rates of IgG secretion to healthy volunteers.

**Figure 2:**
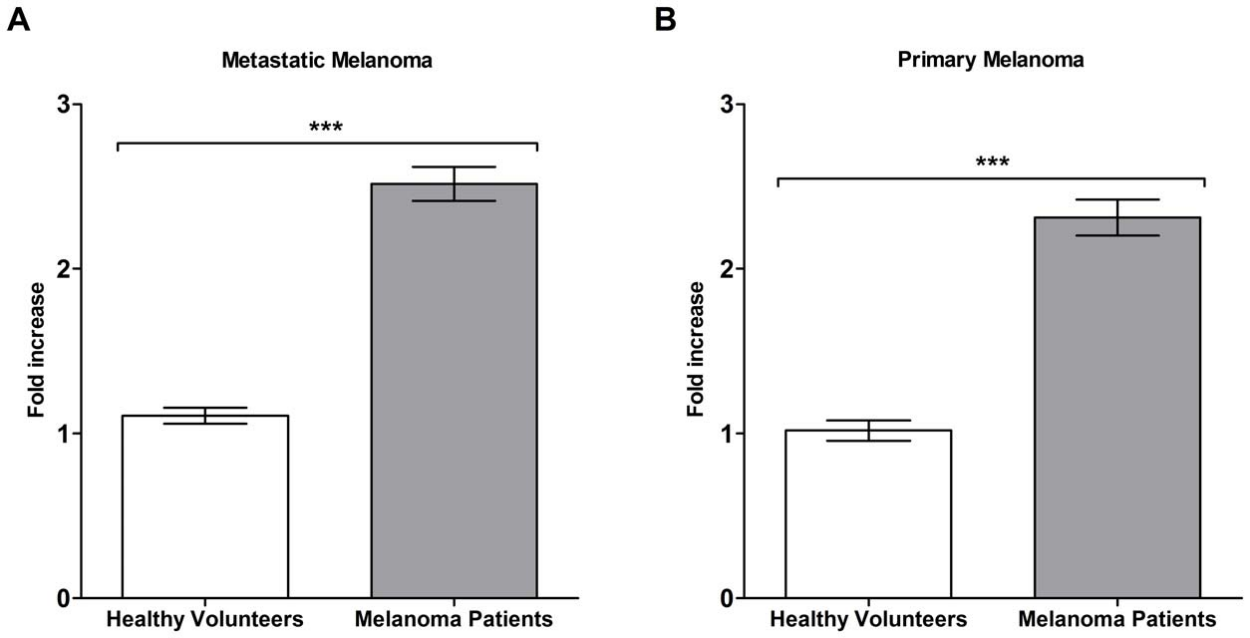
The reactivity of antibodies derived from melanoma patient B cells to primary and metastatic melanoma cells is compared to healthy volunteers. B cell cultures from melanoma patients secreted antibodies that were significantly more reactive against melanoma cell lines compared to healthy volunteers using the cell-based ELISA. Mean reactivity of antibody cultures (n = 600) from 10 melanoma patients to A-375 metastatic melanoma cells (A) and WM-115 primary melanoma cells (B), was compared to 10 healthy volunteer cultures (n = 600) (*P* <0.0001). Fold increase values represent the optical density of each B cell culture relative to the mean optical density of a negative control human IgG antibody. ***  = *P*<0.001. Error bars in figures represent 95% confidence intervals.

We next investigated whether specific antibody responses to melanoma could be detected from circulating B cells of patients and healthy volunteers utilizing our cell-based ELISA. Antibody-secreting B cell cultures from 10 healthy volunteers and 10 patients (n = 4 stage II, n = 4 stage III, and n = 2 stage IV) were evaluated for reactivity to both metastatic and primary melanoma cells relative to non-specific human IgG control (calculated as fold increase above the non-specific human IgG control) employing our cell-based ELISA. We found a significant (*P*<0.0001) increase in the mean reactivity (fold increase) of patient-derived antibody cultures (n = 600) to metastatic melanoma cells (2.5 fold increase, 95% CI =  2.4 to 2.6) compared to antibody cultures (n = 600) derived from healthy volunteers (1.1 fold increase, 95% CI  =  1.1 to 1.2) (Figure 2, A). A significant (*P*<0.0001) increase was also seen in the mean reactivity of patient-derived antibodies to primary melanoma cells (2.3 fold increase, 95% CI  =  2.2 to 2.4) compared to antibodies from healthy volunteers (1.0 fold increase, 95% CI  = 1.0 to 1.1) (Figure 2, B). From this patient cohort, we thus observed a significantly increased reactivity to primary and metastatic melanoma cells, compared to healthy volunteers.

### Antibody Response to Melanoma Decreases with Disease Progression

**Figure 3:**
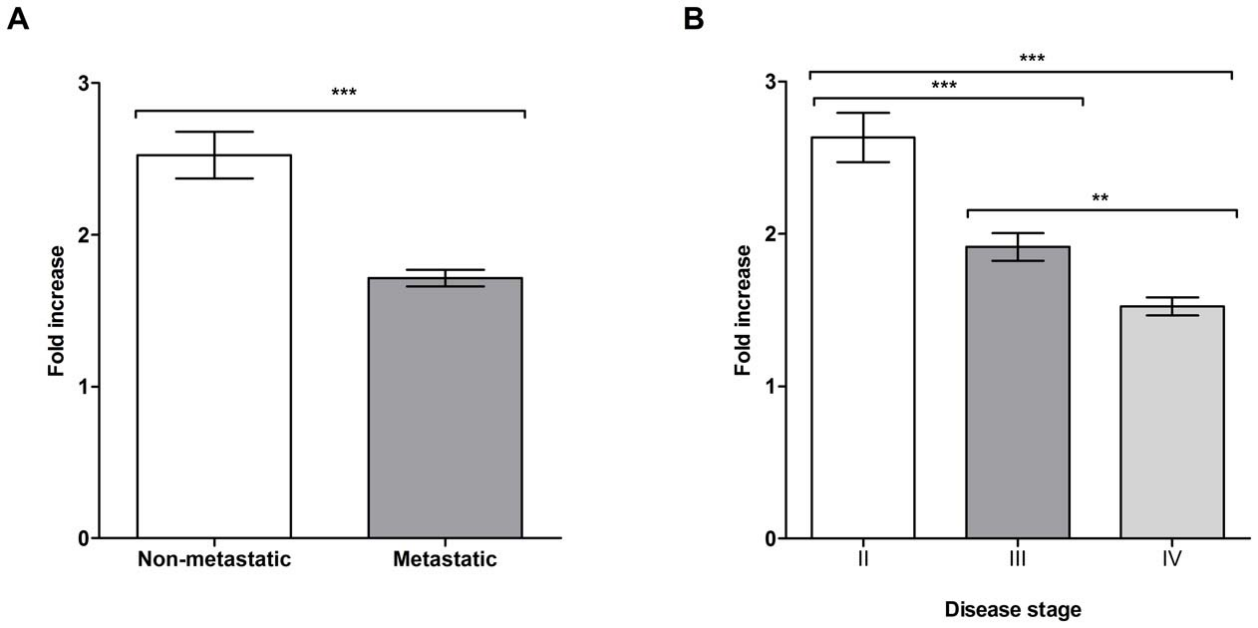
Prevalence of melanoma-reactive antibodies derived from melanoma patient B cell cultures is reduced in advanced disease stages. (A) Comparison of mean antibody culture reactivity to A-375 cells (fold increase relative to negative control human IgG antibody) for patients with localized (non-metastatic, n = 9) and metastatic (n = 12) disease, *P*<0.0001. (B) Mean reactivity to A-375 cells (fold increase relative to negative control antibody) of cultures from patients with stage II (n = 8), III (n = 6) and IV (n = 6) melanoma. Antibody reactivity was determined using a cell-based ELISA. Fold increase values in panels A and B were determined relative to the mean absorbance to a negative control human IgG antibody. ***P<0.001 and ** P =  0.001 to 0.01. Error bars in figures represent 95% confidence intervals.

To examine if antibody responses differ according to disease stage, we studied a cohort of 21 patients diagnosed with stage I, II, III and IV melanoma (Table 1) and evaluated the reactivity of antibody cultures (n = 1,800) from these patients to the metastatic melanoma cell line A-375 utilizing the cell-based ELISA. This patient cohort was almost exclusively Caucasian. Antibody reactivity against melanoma cells was quantified relative to a non-specific human IgG control and measured as fold increase above this negative control. Patients with local (non-metastatic, stages I and II) disease had a significantly (*P*<0.0001) higher mean antibody response (2.6 fold increase, 95% CI  =  2.4 to 2.8) compared to those with confirmed metastatic disease (stages III and IV, 1.7 fold increase, 95% CI  =  1.7 to 1.8) (Figure 3, A). We also found an overall significant reduction (P<0.0001) in the mean reactivity of antibodies secreted in B cell cultures against melanoma cells from stage II (2.8 fold increase, 95% CI  =  2.6 to 3.0, n = 660) to stage III (1.9 fold increase, 95% CI =  1.8 to 2.0, n = 540) and to stage IV patients (1.5 fold increase, 95% CI 1.5 to 1.6, n = 480) (Figure 3, B). The stage I patient was not evaluated since samples from only one patient (n = 120 B cell cultures) from the cohort was available to include in this group. The highest mean antibody reactivity against melanoma cells was observed in Patients 5 and 6 diagnosed with stage II and III melanoma, respectively (Table 1). However, we observed variation in the antibody response among individual patients, with 19 out of 21 patients in our cohort having at least one antibody-producing culture with optical density values 2.5-fold above the negative IgG control (Table 1). These findings suggest that despite the significant reduction in the proportion of tumor-reactive antibody cultures as a function of disease progression, patients from each of stage groups had B cells with antibodies that recognized tumor cells.

**Table 1:** Reactivity of patient-derived antibody-producing B cell cultures to A-375 metastatic melanoma cells.

Stage	Patient ID*	Age	Sex	Ethnicity	Mean fold increase over negative control†	95% CI of mean			Maximum fold increase over negative control	% Mean reactive cultures††
I	9	51	F	Caucasian	1.1	1	to	1	2.8	2
II	4	75	M	Caucasian	2.7	2.6	to	3	4.2	38
II	5	70	M	Caucasian	6.1	5.4	to	7	17	63
II	7	69	M	Caucasian	1.6	1.4	to	2	4.8	3
II	15	49	F	Caucasian	2.6	2.5	to	3	6.2	22
II	1	66	F	Caucasian	1.5	1.4	to	2	6.8	5
II	19	63	F	Caucasian	1.6	1.5	to	2	3.5	82
II	21	38	M	Caucasian	1.3	1.1	to	2	7.7	2
II	20	81	M	Caucasian	2	1.9	to	2	3.7	50
					2.4	2.2	to	3	6.7	33
III	6	67	M	Caucasian	3.2	3	to	3	5.8	14
III	10	54	M	Caucasian	1.8	1.6	to	2	3.6	13
III	16	77	M	Caucasian	1.8	1.6	to	2	3.8	97
III	17	88	M	Caucasian	1.8	1.7	to	2	2.8	40
III	18	68	M	Caucasian	1.3	1.2	to	1	2.8	6
III	8	23	M	Asian	1	0.8	to	1	2.9	8
					1.8	1.7	to	2	3.6	30
IV	11	77	F	Caucasian	1.7	1.5	to	2	3.3	92
IV	12	72	F	Caucasian	1	0.8	to	1	3.5	10
IV	2	66	M	Caucasian	1.8	1.7	to	2	3.1	19
IV	13	55	F	Caucasian	1.5	1.4	to	2	2.6	2
IV	3	51	M	Caucasian	1.8	1.7	to	2	2.7	8
IV	14	31	F	Caucasian	0.7	0.6	to	1	1.3	12
					Mean	1.4	1.3	to	1.5	24

* Patient ID corresponds to patient number in all figures.

† Fold increases values were calculated by dividing the optical density of B cell culture supernatants by the optical density of a non-specific IgG negative control using a cell-based ELISA.

†† % of cultures with absorbance values greater than 75% of a positive control antibody using a cell-based ELISA.

### Estimations of Melanoma-reactive Antibody Frequencies from Patient Peripheral Blood B Cell Cultures

**Figure 4:**
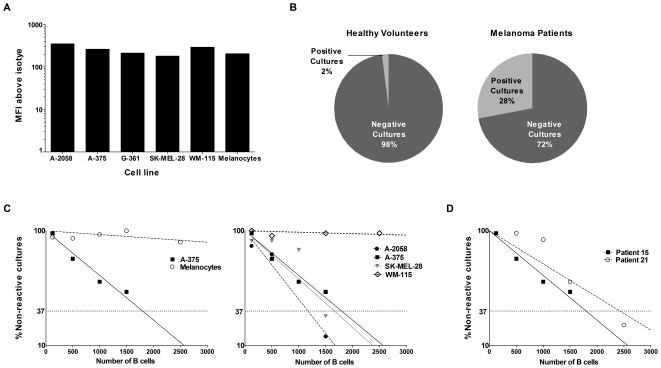
Estimations of the frequency of circulating B cells producing melanoma-reactive antibodies relative to a positive control antibody. (A) Binding of Trastuzumab is consistent across melanoma cell lines and primary human melanocytes evaluated by mean fluorescence intensity (MFI) above isotype control antibody binding for each cell line. (B) Proportion of B cell cultures (n = 1,800) from 21 melanoma patients arising from 500 B cells each that produced antibodies that reacted to metastatic melanoma cells compared to cultures (n = 600) from 10 healthy volunteers. (C) Frequency of B cells producing IgG antibodies able to bind to melanoma cells estimated by limiting dilution analysis for Patient 15 (see Table 1). The frequency of B cells producing antibodies reactive to the cells of interest was approximated according to Poisson distribution, the number of B cells at which 37% of the cultures were non-reactive (dotted horizontal line). Frequencies of tumor-reactive B cells against A-375 melanoma cells versus melanocytes evaluated at the same B cell densities (left) and against four melanoma cell lines (primary WM-115, and metastatic cell lines derived from different anatomic locations, right). (D) Comparison of the frequency of B cells that react to metastatic melanoma cells between two stage II patients, estimated by limiting dilution analysis. For these two patients, frequency was estimated to be 1 in 1,790 B cells (Patient 15) and 1 in 2,430 B cells (Patient 21).

We screened for tumor-reactive antibodies from patient B cell cultures and approximated frequency and specificity of selected cultures to tumor cells. For this we screened B cell culture supernatants against a stringent comparator using a positive control monoclonal antibody, Trastuzumab, that recognizes the HER2/*neu* tumor antigen, expressed on breast cancer cells and on some melanoma cells [38]. Trastuzumab was selected as a positive control because of comparable binding across melanoma cell lines and melanocytes, as shown by mean fluorescence intensities of antibody binding against a range of these cells (Figure 4, A). Previous studies screening for tumor-specific antibodies have selected wells greater than the mean negative control optical density (OD) + three standard deviations as criteria for positive tumor-reactive antibodies [39]. Due to the inherent variability of cell-based assays, and the potential identification of false positive cultures, we chose more stringent criteria for antibody screening, by comparing antibodies produced by B cells to a positive control antibody (> 75% OD of positive control). Based on this antibody selection criteria (>75% OD of positive control), we estimate that 28% of B cell cultures (n = 1,800) derived from 21 patients, each arising from 500 B cells, produced antibodies that recognized metastatic melanoma cells, compared to 2% (n = 600) of cultures derived from 10 healthy volunteers (Figure 4, B). From these 10 healthy volunteers, 2 individuals had one reactive culture (out of 60 cultures), 1 individual had 10 reactive cultures, and the rest of the cohort had no reactive cultures.

From our patient cohort, we can roughly estimate the frequency of B cells that produce an antibody that recognizes melanoma cells under the assumption that a reactive antibody culture, defined as having an OD >75% of the positive control, arises from only a single B cell (1 out of 500 plated per culture). By dividing the total number positive antibody cultures from the patient cohort by the total number of B cells evaluated from the patient cohort by the number of positive antibody cultures, we roughly approximate that from our patient cohort one out of 1,765 B cells produce an antibody that may recognize melanoma cells.

To estimate the frequency of melanoma-reactive antibody-producing B cells in melanoma patients we performed limiting dilution analysis. We selected a stage II patient, who, we predict, may have a high antibody response to melanoma, based on our findings that the antibody responses were highest in this group (Figure 3, B). For this stage II patient (Patient 15, see Table 1) from our limiting dilution analysis assays using the cell-based ELISA, we estimate that one out of 1,790 peripheral blood B cells produces antibodies that bind to A-375 melanoma cells (Figure 4, C). For this same patient, the frequency of B cells producing antibodies that react with melanocytes was also evaluated at the same B cell densities as melanoma cells. We did not observe a comparable patient antibody response to melanocytes as we did to melanoma cells, suggesting a much lower frequency of antibodies that bind to normal cells of the same origin (Figure 4, C left).

Limiting dilution analysis against two additional metastatic (SK-MEL-28, A-2058) and one primary (WM-115) melanoma cell lines for the same patient yielded different but comparable frequencies to A-375 for the metastatic cell lines (SK-MEL-28, 1 out of 1,650 B cells; A-2058, 1 out of 1,170 cells), and a much lower frequency of antibodies that bind to the primary melanoma line WM-115 which was similar to that observed with primary melanocytes (Figure 4, C right). For this patient, the data suggest detectable circulating B cell humoral response frequency against metastatic melanoma cells and lower frequency for normal human melanocytes or primary melanoma cells. To further confirm the frequency observations for the patient-derived circulating B cell repertoire, we performed additional limiting dilution assays for another stage II patient (Patient 21, Table 1). For Patient 21, we estimate 1 out of 2,430 B cells that produces antibodies bind to the same melanoma cell line tested for Patient 15 (Figure 4, D), suggesting lower but comparable frequency to those estimated for B cells from Patient 15. In summary, applying the above methodology, these results suggest that tumor-reactive antibodies from circulating B cells are more frequent in melanoma patients than healthy volunteers and more frequent against a range of metastatic melanoma cells compared to normal melanocytes.

### Screening for Tumor-specific Antibodies and Selection of a Patient-derived Monoclonal Antibody with *In Vitro* Cytotoxicity against Melanoma Cells

**Figure 5:**
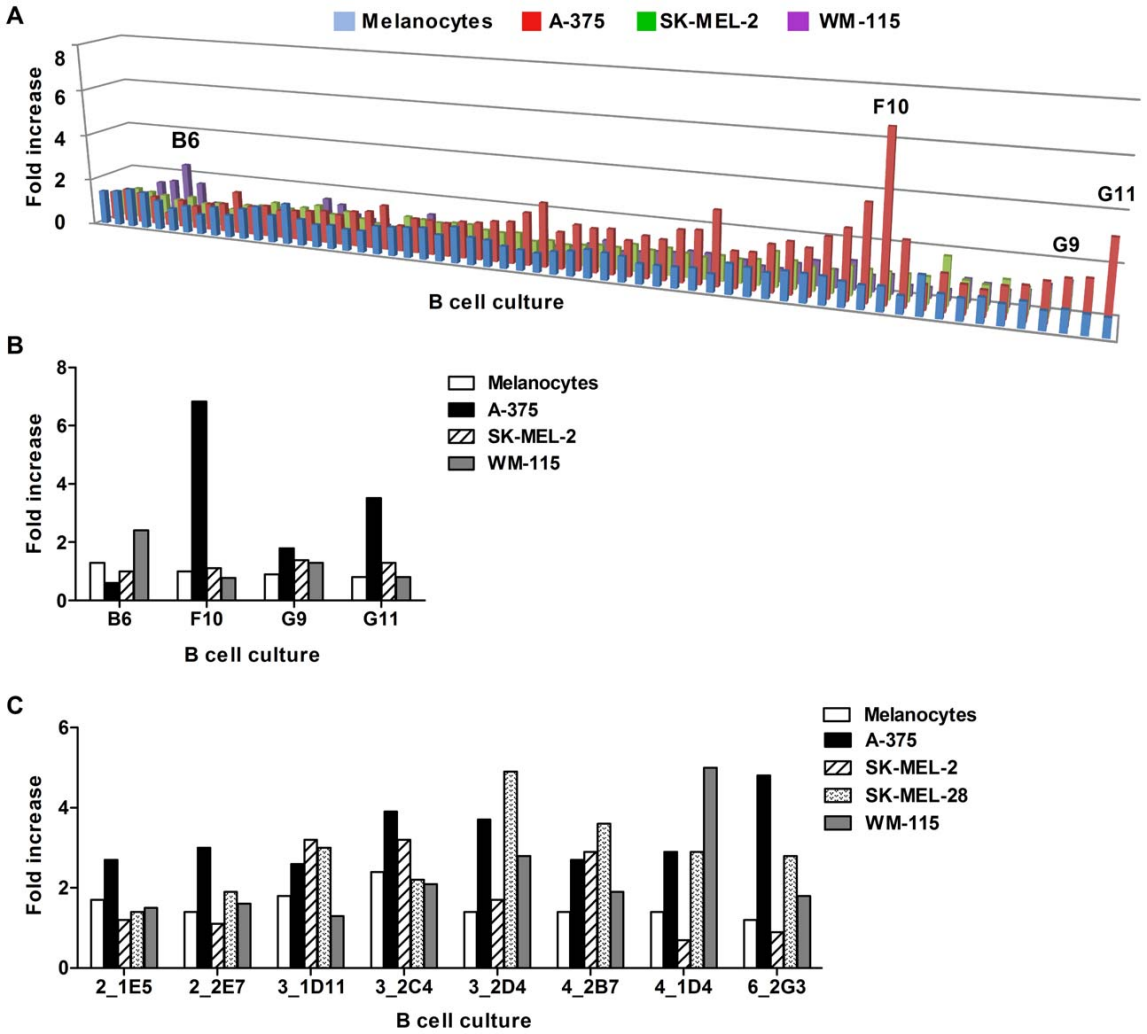
Selection of patient-derived antibodies that recognize melanoma cells. (A) Multiple antibody cultures from one patient (Patient 3, see Table 1) were screened against 3 melanoma cell lines (A-375, SK-MEL-2 and WM-115) and primary melanocytes using the cell-based ELISA. Fold increase values represent optical density (OD) values relative to a negative control antibody. (B) Selected cultures from Patient 3 evaluated in A, which secreted antibodies that bound to the above tumor cell lines are compared to melanocytes. (C) Selected cultures derived from 4 patients were evaluated against 4 melanoma cells lines and compared to melanocytes.

We then selected patient-derived, tumor-specific antibodies in order to further evaluate their reactivity to melanoma cells, and conducted a preliminary assessment of the potential functional capabilities of a patient-derived antibody from this screen. B cell culture wells were selected based on stringent criteria (OD > 75% positive control antibody), using the cell-based ELISA. Tumor specificity of antibody cultures was evaluated by comparing binding of antibodies from these cultures against multiple melanoma cells (A-375, SK-MEL-2, WM-115) versus normal cells (Figure 5, A). We observed multiple antibody cultures with a higher degree of binding to some melanoma cells compared to melanocytes from the same patient (Patient 3, Figure 5, A; a selection of five of these cultures is shown on Figure 5, B). Similar results were obtained when we screened for tumor-specific cultures from different patients against melanoma cells and melanocytes. Positive cultures with different binding patterns against four melanoma cell lines (A-375, SK-MEL-2, SK-MEL-28, WM-115) and primary human melanocytes were detected (selected cultures derived from Patients 2, 3, 4 and 6 are shown as examples in Figure 5, C), reflecting specificity and reactivity of different antibodies to a range of antigens expressed at different levels in a number of melanoma cell lines, and some reactivity to antigens lowly expressed on human melanocytes. Selection of a tumor-positive antibody culture for sub-cloning and limiting dilution was based on degree of reactivity to melanoma cells relative to melanocytes (Figure 5, C).

**Figure 6:**
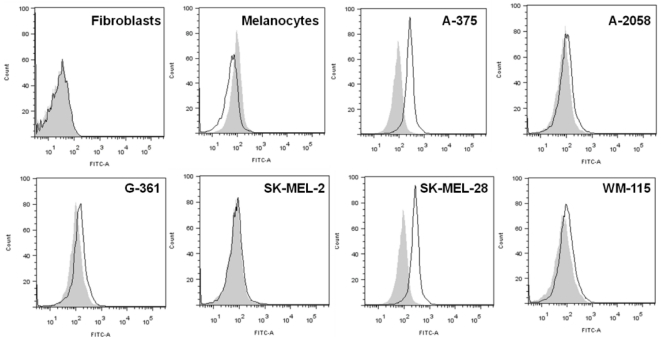
Selection of a patient derived B antibody that recognizes melanoma cells but not melanocytes. A selected tumor-reactive culture from a stage III patient (Patient 6) which secreted antibodies that bound to tumor cell lines and compared to melanocytes by ELISA (Figure 5, C) was sub-cloned, and a monoclonal antibody 6_2G3 was selected and evaluated on live cells by flow cytometry (solid black line histograms) for reactivity to fibroblasts, melanocytes, and 6 melanoma cell lines. IgG isotype controls are shown in shaded grey histograms.

One B cell culture from Patient 6 was selected for further evaluation since cell culture supernatants were observed to have a higher degree of binding to A-375 and SK-MEL-28 cells compared to melanocytes by ELISA (Figure 5, C; right). After limiting dilution of this melanoma-reactive B cell culture, a monoclonal antibody (6_2G3) was further assessed for specificity to 6 melanoma cell lines, melanocytes and fibroblasts by live cell flow cytometry (Figure 6). Since more antibody was available after monoclonal dilution, 2 additional melanoma cell lines along with dermal fibroblasts were evaluated. In concordance with the cell-based ELISA findings (Figure 5, C), the 6_2G3 clone bound to a range of melanoma cell lines, but not to melanocytes (Figure 6). The antibody had no reactivity against primary human dermal fibroblasts. In summary, by evaluating the specificity of antibodies to melanoma cells versus melanocytes and fibroblasts we could identify a melanoma-specific monoclonal antibody clone 6_2G3. While we had limited amounts of monoclonal antibodies our B cell culture supernatants after evaluating melanoma-cell specificity, we were able to conduct a limited functional investigation of this antibody.

**Figure 7:**
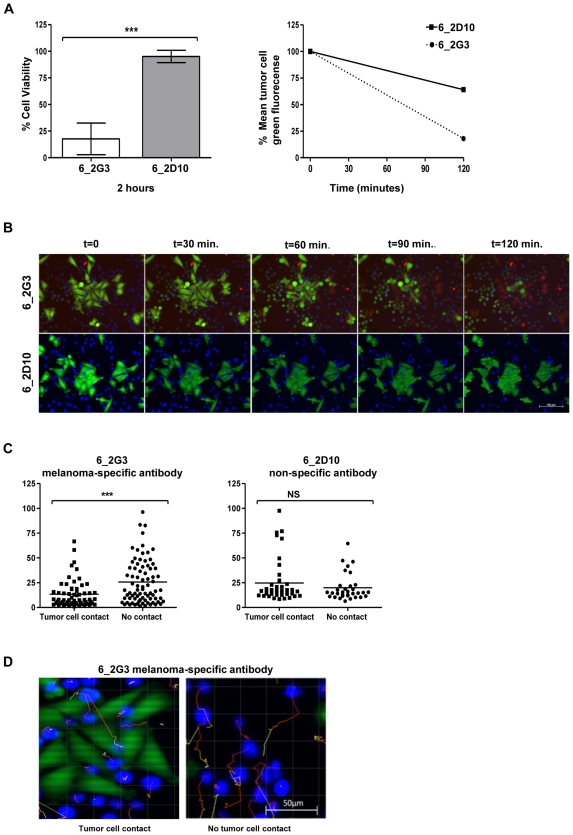
A tumor-specific antibody derived from a patient with melanoma is able to induce tumor cell cytotoxicity. Two monoclonal antibodies were evaluated *in vitro* using a live cell imaging assay: 6_2G3 clone bound to A-375 cells compared to melanocytes; and 6_2D10, a clone also from Patient 6 did not, and served as a negative control antibody for these experiments. (A) Cell viability of A-375 melanoma cells incubated with human U-937 monocytic cells was compared between samples treated with 6_2G3 or 6_2D10 antibody after 2 hours at 37°C (***  = *P*<0.001) (left). Error bars represent 95% confidence intervals. Mean fluorescence intensity of A-375 tumor cells pre-labeled with the live cell dye Calcein AM, and incubated with U-937 cells and antibody 6_2G3 or 6_2D10 was measured at 0 and 120 min time points (right). (B) Fluorescent images of the live cell cytotoxicity assays at 30 minute intervals. Live Calcein AM-labeled melanoma tumor cells (green) were incubated with 6_2G3 or 6_2D10 antibody and U-937 cells (blue) and cell death was evaluated (red). Incorporation of Ethidium homodimer-1 (incorporation of red into tumor cells) was observed with 6_2G3 but not 6_2D10 (magnification 20x, Scale bar: 100 µm). (C) Movement of U-937 cells tracked and measured over two hours was compared for cells in contact and those not in contact with tumor cells (***  = *P*<0.001for 6_2G3 and *P* = 0.3 for 6_2D10 antibody) (upper panel). (D) Images of U-937 movement in tumor cell cultures treated with 6_2G3, tracked for cells in contact (left) and cells not in contact with tumor cells (right). Movement is indicated by tracking lines (red to yellow) from the original position of U-937 cells at t = 0 to t = 2 hours (magnification 20x, Scale bar: 50 µm).

Using clone 6_2G3, we wished to assess whether a patient-derived antibody has potential cytotoxic activity against tumor cells. We tested the tumor cell killing potential of this antibody using a real-time live-dead cell cytotoxicity assay using as targets metastatic melanoma cells recognized by this clone (Figure 7 & Supporting Videos S1 and S2). In these experiments, U-937 human monocytic cells which express Fcγ receptors served as effector cells [40] and A-375 melanoma cells were used as target cells to evaluate antibody-dependent cellular cytotoxicity (ADCC) of tumor cells mediated by patient-derived IgG antibodies. We tested two monoclonal antibodies, both derived from Patient 6 (Table 1): (1) the 6_2G3 antibody, which bound to A-375 cells and not melanocytes and (2) the 6_2D10 antibody, which did not bind to A-375 cells or melanocytes in the cell based ELISA prior to limiting dilution, which served as a non-tumor-reactive control (Figure 7).

After 2 hours in culture, 18% (95% CI  =  -5 to 41%) of tumor cells given the melanoma-specific antibody were viable, compared to 95% (95% CI  =  86 to 104%) of the tumor cells given the non-melanoma specific antibody (*P*<0.0001) (Figure 7, A, left). Relative to tumor cell fluorescence at the start of the assay, mean green/live tumor cell fluorescent intensity was reduced to 64% for the non-tumor specific antibody (6_2D10) compared to 18% for the tumor-specific antibody (6_2G3) (Figure 7, A, right). These results highlight the potential of a patient derived tumor-specific antibody to kill tumor cells by antibody-dependent cell cytotoxicity (Figure 7, B and see Videos S1 and S2). For tumor cells treated with the tumor-specific 6_2G3 antibody, we also observed a significant (*P*  = 0.0002) reduction in the movement of monocytic effector cells in contact with tumor (13 µm, 95% CI  =  10 to 17 µm) compared to effector cells not in contact with tumor (26 µm, 95% CI =  21 to 31 µm) (Figure 7, C and D). Using the 6_2D10 non-specific antibody, no significant (*P* = 0.3) difference was observed for the movement of effector cells not in contact with tumor cells (20 µm, 95% CI =  15 to 25 µm) compared to those in contact with tumor cells (25 µm, 95% CI = 18 to 31). With this example, we demonstrate that a patient-derived tumor-specific antibody is capable of engaging immune effector cells in antibody-dependent cellular cytotoxicity against tumor cells. Taken together, these data suggest that systemic melanoma-specific mature B cell responses may be present in patients with melanoma and may harbor the potential to be activated against cancer cells.

## Discussion

We describe an approach to study the circulating B cell-derived humoral immune response to cancer and apply this to detect tumor-specific IgG antibodies from melanoma patient B cells. This strategy has the potential to be applied to any type of cancer. Findings presented herein complement previous serological studies, providing added insight into the mature systemic B cell response to melanoma.

As a first step to evaluate the tumor reactivity and specificity of patient B cell-derived IgG antibodies, we developed a medium-throughput cell-based ELISA with melanoma cells to detect antibodies against tumor cell surface antigens (Figure 1). Cells were allowed to grow and adhere on to 96-well plates prior to being preserved by a light fixative (0.5% formaldehyde). While preserving the cells and allowing for storage and access to multiple plates at any one time, light fixation with formalin allows preservation of potentially-antigenic epitopes on the surface of target cells. Previous studies have reported cell-based ELISA methods to identify tumor-specific antibodies in melanoma [39], [41], where tumor cells were preserved using strong fixatives such as glutaraldehyde, known to potentially mask antigenic epitopes, thus compromising the recognition of antigens by antibodies [42]. Furthermore, the specificity and sensitivity of such methods has not been reported using antibodies against known cell surface antigens. Although many intracellular melanoma associated antigens have been described (tyrosinase, TRP-1, TRP-2, gp100/pmel17), most are also expressed by normal melanocytes, only a few defined cell surface antigens such as the High Molecular Weight Melanoma-Associated Antigen (HMW-MAA) are reported to be expressed on the surface of melanoma cells, and other antigens show heterogeneous expression among patients [43], [44]. Thus, this ELISA constitutes an attractive tool to evaluate broad responses to any naturally-expressed antigens on the surface of melanoma cells and melanocytes in this context. This screening methodology has additional potential advantages. Unlike assays screening against a single recombinant antigen or antigenic epitope, our method enables the evaluation of antibody repertoires of patients against a multitude of cell surface antigens in their native confirmation on the surface of both primary and metastatic melanoma cells and also melanocytes, providing more comprehensive information on the broad prevalence of tumor-reactive and tumor-specific antibodies. Previous studies have shown concordance of cell line-associated antigens with antigens expressed on corresponding tumors, making them a suitable platform for tumor-reactive antibody screening [26], [45]. Thus, cell lines provide a promising alternative source of multiple tumor antigens in the absence of multiple well-defined, highly expressed, and readily available recombinant antigens. Unlike flow cytometric evaluations, the cell-based ELISA does not require the use of proteolytic enzymes such as trypsin, therefore better preserving cell surface antigens. Plates of target cells can be prepared, fixed and frozen in batches, thus allowing for higher throughput screening for tumor cell-reactive antibodies. It can be applied to evaluate > 300 culture supernatants against cell lines within a few hours. In principle, numerous ELISA plates for screening a range of cell lines with multiple supernatant samples can be processed simultaneously. Additionally, this methodology may be a potential tool for immunomonitoring tumor-specific humoral responses to therapies; selecting patients most likely to benefit from immunotherapy; or as a prognostic factor in linking tumor-reactive humoral responses to clinical outcomes. This assay may also be utilized to detect surface antigens in a range of cell types, and thus may be adapted to monitor the B cell-derived antibody repertoire in different disease contexts.

In agreement with a recent report [46], we also observed a reduction in the peripheral blood memory B cell compartment of metastatic melanoma patients. We measured a reduction of the CD27+ subset of memory B cells in patients with both metastatic and non-metastatic melanoma compared to healthy volunteers (Figure S2). Despite the reduction of circulating memory B cells in our cohort, patient-derived B cells were capable of secreting high amounts of IgG antibodies when activated *in vitro* with a TLR 9 agonist, with comparable antibody production to B cells from healthy individuals, and a high percentage of patient- and healthy volunteer-derived B cells expressed IgG antibodies within a few days in culture (80% of B cells from three patients with melanoma, Figure S2). Thus, while a reduced memory B compartment has been reported in cancer patients, we show that a melanoma-reactive portion of this compartment remains in our patient cohort.

We demonstrated a high prevalence of melanoma patient-derived antibodies produced by circulating B cells in cancer patients that recognize melanoma cell lines (Figure 2). We observed that B cell culture supernatants from different patients displayed differential binding to each cell line, which reflects specificity and reactivity of different antibodies to a range of antigens expressed at different levels in a number of melanoma cell lines; these may also reflect binding to some antigens lowly expressed on human melanocytes (Figure 4 and Table 1). Melanoma patients had a high percentage of melanoma-reactive antibody-producing B cell cultures, significantly higher than those from healthy volunteer-derived B cell cultures (Figure 2), with 28% of melanoma patient-derived B cell cultures recognizing melanoma cells, compared to 2% of cultures from healthy volunteers (Figure 4). Limiting dilution analyses of reactivity against melanoma cells versus normal melanocytes provided further evidence in support of the presence and frequency of tumor-reactive B cells in patient blood (Figure 4). For one stage II patient evaluated, the data indicate that metastatic melanoma cells are recognized by a higher proportion of B cells (estimated on average ∼ 1 in 2,000 mature B cells) compared to primary melanoma cells or melanocytes, although in a cohort of 10 patients we measured reactivity of B cell cultures to both metastatic and primary melanoma cell lines (Figure 2). Taking into consideration the expected variability in immune responses among patients, and the array of tumor antigens these patients may be exposed to, the observations that B cells from two patients with stage II melanoma yielded comparable reactivity to metastatic melanoma cells (estimated 1 in 1,790 for Patient 15, and 1 in 2,430 B cells for Patient 21) indicate the presence and support the prevalence of a circulating melanoma-reactive B cell compartment.

While tumor-reactive antibodies were detected from most melanoma patients studied, antibody responses derived from circulating B cells against melanoma cells decreased with more advanced disease stages (Figure 3). Previous serological studies report serum-resident antibodies against tumor cells in melanoma patients, with some evidence that serum antibodies are diminished in patients with advanced disease [25], [47]. It was unclear whether this was a consequence of the sequestering of antibodies into tumors with increasing tumor burden in these patients. Our findings provide further insight by demonstrating the presence of a circulating long-term mature B cell response to cancer at all disease stages, against a broad range of naturally expressed antigens on the surface of primary and metastatic tumor cells. We also report decreased frequency of tumor-reactive antibody-producing B cells with advanced disease, thus supporting the premise that mechanisms of immune tolerance rather than adsorption of antibodies into tumors in advanced disease setting may also explain these reductions. One limitation may arise from screening for antibodies against mostly metastatic melanoma cells. It is possible that our observations may reflect reactivity to antigens present in primary disease, which may be preserved or upregulated in advanced disease setting. While our findings may not account for reactivity to tumor antigens that are lost with disease progression, this reduced reactivity to melanoma we observed may imply weakened immune responses to a subset of antigens on the surface of melanoma cells. Another explanation for these observations may be that with advanced disease, mature circulating B cells home into increasing tumor sites, thus reducing the circulating tumor-reactive B cell compartment in these patients. Future studies aimed at monitoring local B cell responses in tumors may provide further clues into the dynamics of mature B cell responses at the systemic and local levels in cancer. Thus, despite well-known weakened host immune response with disease progression [48], we were able to detect melanoma-reactive antibodies from patient circulating B cells, implying that although mature humoral immune responses are weakened, responses in the form of mature memory B cells may persist. However, further work elucidating potential immunomodulatory roles of B cells and other immune cells in cancer, including the production of IL-10 by B cell population subsets [49], merits consideration.

Although we report the presence of anti-tumor antibodies produced by patient memory B cells, and these cells were stimulated *ex vivo* to secrete antibodies, it is not clear whether tumor antigen-reactive B cells are activated in patients to secrete antibodies or whether these humoral responses are capable of exerting any beneficial anti-tumoral activities in the same patients *in vivo*. In the 21 patient cohort at different disease stages in this study, we were not able to draw any conclusions regarding the relationship between tumor cell reactivity and clinical disease progression in the short term (6 months to 2 year follow up) or associations with any particular disease treatment regimes. However, monitoring mature memory B cells and their antibody repertoires together with clinical outcomes in patients over a long period of time may help identify any correlations between melanoma-reactive mature memory B cell responses and disease progression. Additionally, future studies may help identify particular components of the humoral response which may hold clinical relevance, and elucidate the potential merits of monitoring these responses in relation to therapies, or of evaluating humoral responses as a prognostic factor to clinical outcomes.

An important question therefore relates to whether patient-derived mature B cell responses have any functional capability to potently activate immune effector cells against cancer. For this, we measured the capacity of one antibody clone to kill tumor cells. Antibody clone 6_2G3 derived from a patient with stage III disease (Patient 6, Table 1) was not observed to bind to fibroblasts or melanocytes, but bound to a proportion of melanoma cell lines tested (Figure 5). Antibodies against tumor-associated antigens can attack tumor cells via a number of mechanisms including induction of apoptosis in tumor cells and engaging Fc receptors on immune cells [50], [51], [52], [53]. Antibodies approved for the treatment of cancer have been shown to function through one or more of these mechanisms [54], [55]. While our strategy yields fully human monoclonal antibodies in a matter of a few months, we were limited in the amount of antibody we could produce from the B cells to perform functional studies and evaluate reactivity to patient-derived melanoma tumors. However, we had sufficient quantity to evaluate whether a patient-derived melanoma tumor-specific monoclonal antibody could mediate antibody dependent cellular cytotoxicity (ADCC) in the presence of monocytic effector cells and tumor cells using a real-time live cell imaging assay. We show that the tumor-specific 6_2G3 clone is capable of mediating ADCC *in vitro* and additionally measured the restricted movement of monocytic effector cells once in contact with tumor-specific antibody-coated tumor cells, providing further evidence of ADCC (Figure 6). These preliminary assessments provide a promising clue that a potentially active mature B cell response against melanoma may be present in patients. An example of this possibility was recently reported by Yuan et al. who demonstrated that administration of the anti-CTLA-4 antibody ipilimumab led to serological enhancement of antibodies to the testis antigen NY -ESO-1 in patients who responded to the antibody therapy [56]. It is therefore conceivable that the mature B cell compartment could be enhanced with immunotherapeutic approaches, and that monitoring humoral responses to therapeutics may have clinical relevance.

Harnessing the cancer-specific antibody repertoire of cancer patients using the methodology described herein may also potentially offer an alternate strategy to yield IgG antibodies against cancer antigens. Recent advances reported by Traggiai et al., evaluating monoclonal antibodies from human memory B cells have yielded fully-human virus-neutralizing antibodies of therapeutic relevance for infectious diseases and have contributed to the dissection of humoral memory responses to vaccinations [35], [57], [58]. Here, we focus on B cells from cancer patients such as melanoma patients, analyze systemic humoral responses to cancer and demonstrate the presence of tumor-reactive and tumor-specific antibodies. This approach may offer an advantage over other approaches such as phage display in that it yields *in vivo* affinity-matured human antibodies with naturally paired heavy and light chains. The patient-derived monoclonal antibody 6_2G3 bound to 2 out of 6 of the melanoma cell lines evaluated compared to melanocytes, suggesting that this antibody may be against a protein over-expressed or mutated on the surface of cancer cells. In light of the efficacy of Trastuzumab, against the HER2/*neu* antigen expressed on 20–30% of breast cancers, as a clinically-validated therapeutic tool for the treatment of an equivalent proportion of breast cancer patients [59], selection of antibodies that bind to a portion of cell lines may merit further characterization. Although the clinical significance of mature memory B cells expressing antibodies that recognize tumor cells in patients remains to be elucidated, antibodies derived from these cells, introduced by passive immunotherapy in therapeutically-relevant doses, such as those used for Trastuzumab to patients with breast cancer, merit investigation for any potential relevance in melanoma. Other potential future benefits of screening patient-derived B cells from tumor-reactive antibodies may be identification of novel cell surface tumor antigens. Future evaluations of clone 6_2G3 will include sequence analysis and expression cloning to allow for further analyses of specificity to melanoma tumors, antigen identification, and for thorough functional assessments.

These data provide additional understanding of the mature B cell response to melanoma by evaluating antibodies derived from circulating B cells of cancer patients. The prevalence of mature humoral responses against cancer cells in patients, as well as the capacity of a patient-derived antibody to activate effector cells against melanoma cells indicate the potential functional significance of the humoral immune response against cancer.

## Materials and Methods

### Ethics Statement

Specimens from patients and healthy volunteers were collected with informed written consent. The work was conducted in strict accordance with study design approved by the Guy’s Research Ethics Committee, St. Thomas’ Hospital, London, UK.

### Study Subjects and Isolation and Culture of Peripheral Blood Human B Cells

After obtaining informed consent, peripheral blood was isolated from healthy volunteers (n = 10) and from patients with melanoma (n = 21). Patients were staged and classified according to the American Joint Committee on Cancer Melanoma Staging and Classification criteria [60]. B cells were isolated by negative selection using RosetteSep® B cell enrichment cocktail (Stem Cell Technologies, Vancouver, Canada) according to the manufacturer's instructions. B cell purity was assessed by flow cytometry by staining for mature B cells (CD22), T cells (CD3), monocytes (CD14) and plasmacytoid dendritic cells (BDCA3) using fluorescently-labeled monoclonal antibodies, all from BD Biosciences, Oxford, UK (Figure S1). Flow cytometry experiments were conducted with either the FACSAria or FACSCanto (BD Biosciences) and flow cytometric data were analyzed using Flow Jo (Tree Star, Ashland, OR).

B cells were plated at 500 cells per well on 96 well U-bottom microplates (Nunc, Rochester, NY) along with 3x10^4^ cells per well of irradiated (30 Gy) autologous PBMCs, obtained by Ficoll centrifugation, as feeder cells. B cells were grown in RPMI-1640 medium obtained from Gibco (Invitrogen, Carlsbad, CA) supplemented with 10% fetal calf serum, 1% penicillin-streptomycin, 2.5 ng/mL TLR9 ligand CpG 2006 ODN (Operon, Ebersberg, Germany), and 30% supernatant of Epstein Barr Virus (EBV) producing B95-8 cells [35]. For each patient evaluated, 60-120 B cell cultures originating from 500 B cells each were established, and cultures were grown in 200 µL per well volumes. After 18 days, supernatant (40 µL) from each culture well was screened individually for tumor-specific antibodies and selected B cultures were sub-cloned by limiting dilution to derive monoclonal cultures. We plated B cells at 1 cell/well in the presence of 3x10^4^ autologous 30 Gy irradiated autologous PBMC stimulated with 2.5 ng/mL CpG 2006 ODN.

### Cell Lines and Culture

Human dermal fibroblasts were a gift from Dr. Christian Hundhausen, King’s College London, UK. All other cell lines used were obtained from the American Type Culture Collection [ATCC] (Manassas, VA). Cell lines were used to identify tumor-reactive antibodies and to test for cytotoxic activity of antibodies. Media used for cell lines A-375 (CRL-1619), A-2058 (CRL-11147), G-361 (CRL-1424)**,** SK-MEL-2 (HTB-68), SK-MEL-28 (HTB-72), SK-BR-3 (HTB-30), U-937 (CRL-1593.2) and WM-115 (CRL-1675) were obtained from Gibco and supplemented with 10% fetal calf serum and 1% penicillin-streptomycin. The human metastatic melanoma cell lines A-375 and A-2058 were grown in Dulbecco’s Modified Eagle’s Medium. The human melanoma cell line derived from primary melanoma tissue, WM-115, and the metastatic melanoma cell lines SK-MEL-2 and SK-MEL-28 were grown in Eagle’s Minimum Essential Medium. The human metastatic melanoma cell line G-361, and the human mammary carcinoma cell line SK-BR-3, which expresses the Human Epidermal Growth Factor Receptor 2 (HER2/*neu*), were grown in McCoy's medium. The Fc receptor-expressing monocytic-like U-937 cell line was grown in RMPI-1640 medium. Primary human melanocytes (ATCC, PCS-2000-012) were grown in Dermal Cell Basal Medium (ATCC) and supplemented with the Melanocyte Growth Kit (ATCC). Human fibroblasts were grown in Medium 106 (Invitrogen) and supplemented with Low Serum Growth Supplement (Invitrogen).

### Detection of Antibodies Bound to Tumor Cell Surface Proteins by Immunocytochemistry and Flow Cytometry

Qualitative detection of tumor-specific antibodies by immunocytochemistry was performed by centrifugation of 2×10^5^ cells at 300g using a Shandon Cytospin® 4 Cytocentrifuge (Thermo Fisher Scientific, Waltham, MA) onto glass slides. Cells were fixed in 0.5% formalin and antibodies, such as those recognizing the human High Molecular Weight Melanoma-Associated Antigen (anti-HMW-MAA clone LHM2, Invitrogen, Carlsbad, CA), were incubated overnight at 4°C and detected following a 2 hour incubation at 4°C with a horseradish peroxidase-conjugated anti-IgG Fc-specific antibody (1:100 dilution in Tris Buffered Saline, Sigma, Dorset, UK). Slides were stained with DAB chromogenic substrate (DAKO, Ely, UK) for 5 minutes, washed and counterstained with Mayer’s hematoxlin (Merck, Darmstadt, Germany) for one minute, dehydrated and mounted in DPX mountant (Sigma) prior to assessments.

Antibodies bound to cell surface antigens were also detected on live cells by flow cytometry. Adherent cells were detached using StemPro® Accutase® cell disassociation solution (Gibco) and incubated at 2×10^5^ cells per sample with antibody, isotype control or cell culture supernatants for 30 minutes at 4°C. Antibodies bound to cells were detected using a FITC-conjugated anti-IgG Fc-specific antibody (Jackson ImmunoResearch). The binding of tumor-specific antibodies to cells was compared to an excess of isotype control IgG_1_ antibody (Jackson ImmunoResearch). Binding of Trastuzumab across melanoma cell lines and primary human melanocytes was evaluated by subtracting the mean fluorescence intensity (MFI) values of equal amounts of isotype control. Evaluations are representative of three experiments.

### Development of a Cell-based ELISA to Detect Tumor-specific Antibodies

We developed and employed a novel cell-based ELISA to identify melanoma-reactive antibodies. Adherent cells of interest were plated at 3×10^5^ cells per in 200 µL of appropriate media well on 96-well flat bottom tissue culture plates (Corning, Corning, NY) and were grown in a monolayer at 37°C and 5% CO_2_ to 80-100% confluence. Cells were then lightly fixed in 0.5% formaldehyde/Hank’s Buffered Salt Solution. Plates were then wrapped in foil and placed in a -80°C freezer until the day of the assay. On the day of the assay, plates were thawed for 30 minutes, washed 3 times with PBS and then blocked with a 5% non-fat milk/PBS solution for 2 hours. After removal of the blocking solution, 50 µL of culture supernatants or tumor-specific antibodies were diluted 1:2 in 1% non-fat milk/PBS solution and then added to each well, and plates were incubated for 90 minutes at room temperature on an orbital shaker. Plates were then washed 4 times with PBS/0.05%Tween (PBS-T). The binding of antibodies to cell surface proteins was detected following a 45 minute incubation with a goat anti-human horseradish peroxidase-labeled F(ab)’_2_ Fc-specific antibody (Jackson ImmunoResearch, West Grove, PA) diluted 1:250 in 1% milk/PBS-T at room temperature on an orbital shaker. Wells were then washed 4 times with PBS-T. The color reaction was developed for 15 minutes with OPD (Sigma) and OD was measured in an ELISA reader (BMG Labtech, Offenbury, Germany) at 492 nm (reference wavelength, 650 nm). Each plate contained triplicate wells of a positive control antibody, Trastuzumab (Genentech, South San Francisco, CA), and a negative control antibody, non-specific human IgG_1_ (Jackson Immunoresearch) at a concentration of 250 ng/mL both diluted in RPMI-1640 media supplemented with 10% fetal calf serum. Binding of Trastuzumab to cells and background OD values for the negative non-specific human IgG control antibody formed the criteria for inclusion of readouts in the study. Since we were limited by the volume of culture supernatants for each culture, assays were repeated only when sufficient culture supernatants were available to confirm reproducibility of readouts.

### Criteria for Evaluating Antibody Responses to Melanoma Using the Cell-based ELISA

Patient and healthy volunteer antibody responses were assessed using the cell-based ELISA. We evaluated the reactivity of the supernatant from each B cell culture to tumor cells relative to negative and positive control antibodies. In order to compare anti-tumor antibody responses to metastatic and primary melanoma cells between patients and healthy volunteers, and among patient groups, optical densities (OD) were normalized using the following formula:
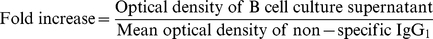
Additionally, this calculation was used to normalize ELISA results among multiple melanoma cell lines and primary melanocytes in order to evaluate the tumor specificity of antibodies.

To evaluate the presence and estimate the frequency of tumor-reactive antibodies, we selected wells with OD values above 75% of the OD of the positive control antibody. To compare the percentage of positive cultures across patients, OD values were normalized against the positive control. For these evaluations, the mean positive control OD was assigned a relative absorbance of 1 for each plate and B cell cultures were converted from OD units to relative absorbance, and culture wells with relative absorbance values greater than 0.75 to melanoma cells but not melanocytes were selected. These criteria were also applied in limiting dilution assays to estimate the percentage of non-reactive B cell culture well. In these limiting dilution assays, B cells were plated at different densities (ranging from 125 to 2,500 B cells) and the percentage of non-reactive cultures was calculated for different patients and cell lines as a way to approximate the frequency of B cells producing melanoma-reactive antibodies using Poisson distribution.

### Live Cell Imaging Assays to Measure Antibody-Dependent Cellular Cytotoxicity

The tumor-killing potential of 2 patient-derived monoclonal antibodies was assessed: one tumor-specific antibody (6_2G3), and another antibody that did not recognize tumor cells (6_2D10), both derived from the same patient (Patient 6). Both antibodies were simultaneously evaluated using a three-color fluorescent live cell imaging cytotoxicity assay. A-375 cells were plated overnight at 2x10^5^ cells per well on 6-well culture plates (Corning). Using a LIVE/DEAD^®^ Viability/Cytotoxicity kit (Molecular Probes, Eugene, OR) live tumor cells were labeled with 2µM of Calcein AM 30 minutes prior to cytotoxicity assays, washed in RPMI 1640 supplemented with 10% FCS and 1% penicillin streptomycin, and re-suspended in media containing 4 µM Ethidium homodimer-1. Ethidium homodimer-1 incorporates into the DNA of dead cells and served as a label for cell death in this assay. U-937 monocytic cells expressing Fcγ receptors were used as immune effector cells at a ratio of 3:1 (effectors: tumor cells) [40]. U-937 monocytes were incubated with the 6_2G3 or 6_2D10 antibody for 30 minutes, stained with the CellTracker™ Blue dye (4-chloromethyl-7-hydroxycoumarin) (Molecular Probes), washed and added to the Calcein AM-labelled tumor cell cultures containing Ethidium homodimer-1. Samples were incubated and images were captured every 5 minutes for two hours in a humidified temperature controlled chamber using a Zeiss Axiovert microscope equipped with a LD-Plan-Neofluar 20x/0.4 Korr/Ph2 objective and AxioVision software system (Carl Zeiss, Jena, Germany). Following incubation, fluorescent intensities of Calcein AM-positive live tumor cells, as well as incorporation of Ethidium homodimer-1 into cells were measured and cell death was assessed with NIS-Elements BR 3 software (Nikon). The movement of effector cells in the cultures was tracked and analyzed using IMARIS software (Bitplane, Zurich, Switzerland).

### Statistical Methods

Descriptive statistics were generated to examine the distribution of melanoma-reactive B cell cultures from each patient including the mean, 95% confidence interval and maximum reactivity to melanoma cells. A two-sided Student’s *t* test was used to compare the mean reactivity of antibody cultures derived from melanoma patients to healthy volunteers to primary or metastatic melanoma cell lines and to compare antibody responses between patients with non-metastatic and metastatic disease. A one-way ANOVA was used to compare antibody reactivity to a metastatic melanoma cell line among B cell cultures derived from patients with stage II, III and IV disease with a Tukey’s post hoc comparison test. A two-sided Student’s *t* test was used to compare antibody-mediated tumor cell killing between tumor-specific and non-specific monoclonal antibodies derived from the same patient. A two-sided Student’s *t* test was also employed to compare the movement of immune effector cells, pre-incubated with antibodies, in contact with tumor cells to the movement of immune cells not in contact with tumor cells. All statistical analyses were performed using GraphPad Prism software (version 5.03, GraphPad, San Diego, CA) and error bars in all figures represent 95% confidence intervals.

## Supporting Information

Figure S1 Schematic of cell-based ELISA used to detect antibodies against tumor cell antigens.
(TIF)

Figure S2 Secretion of IgG antibodies from peripheral blood B cells derived from patients and healthy volunteers.
(TIF)

Video S1 Real-time live-cell cytotoxicity assay for the 6_2G3 melanoma-specific antibody.
(AVI)

Video S2 Real-time live-cell cytotoxicity assay for the 6_2D10 non-melanoma-specific antibody (negative control).
(AVI)
Videos S1 and S2
These video files show our real-time cytotoxic assays. Video S1 shows real-time functional data of the 6_2G3 melanoma-specific patient derived antibody which was observed to kill melanoma cells. Video S2 shows the identical assays shown in Video S1 using a non-melanoma specific antibody derived from the same patient, as a negative control. In these assays, live tumor cells are labeled in green (live cell dye), U-937 monocytic cells are labeled in blue, and cell death is indicated by the incorporation of red (Ethidium homodimer-1 incorporation). Frames from these videos are also displayed in Figure 7, B.
