# Supplementary figures and images for: Monitoring the Systemic Human Memory B Cell Compartment of Melanoma Patients for Anti-Tumor IgG Antibodies
Source: PLoS One. 2011 Apr 29;6(4):e19330. doi: 10.1371/journal.pone.0019330 (PMC3084832; doi:10.1371/journal.pone.0019330)

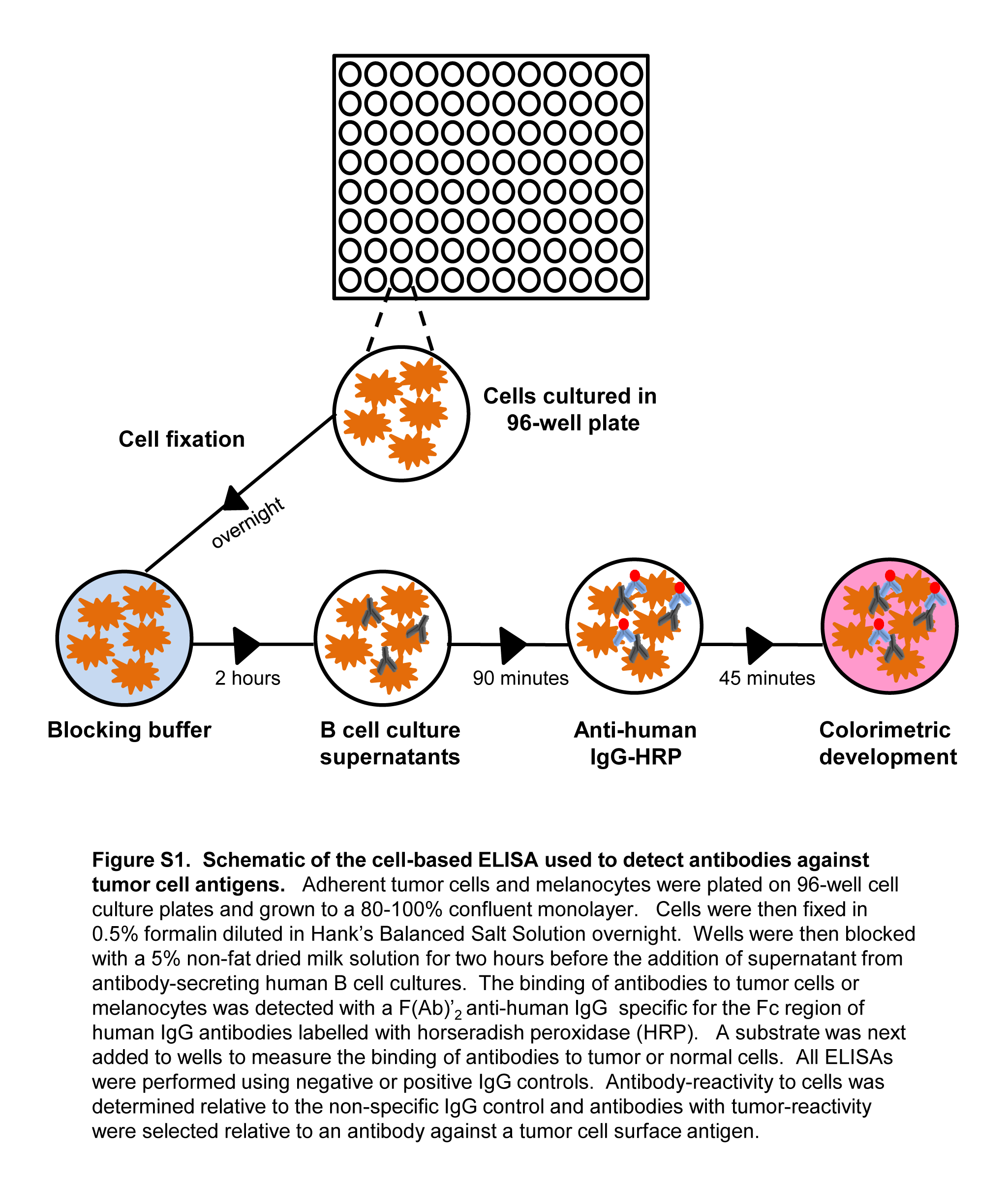

Supplement: Figure S1 — Schematic of cell-based ELISA used to detect antibodies against tumor cell antigens. (TIF) [file pone.0019330.s001.tif]

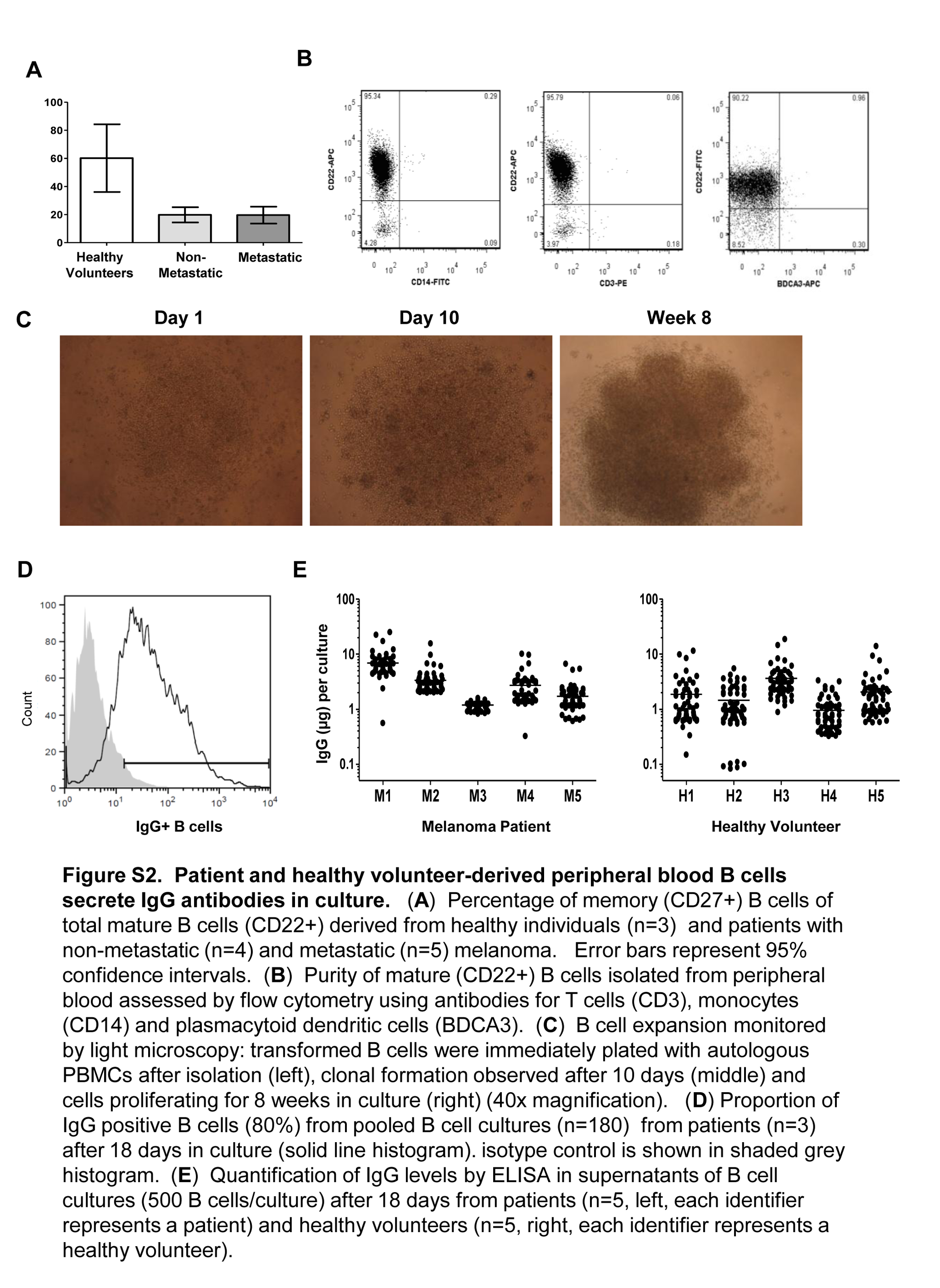

Supplement: Figure S2 — Secretion of IgG antibodies from peripheral blood B cells derived from patients and healthy volunteers. (TIF) [file pone.0019330.s002.tif]
